# Patients with sporadic FTLD exhibit similar increases in lysosomal proteins and storage material as patients with FTD due to *GRN* mutations

**DOI:** 10.1186/s40478-023-01571-4

**Published:** 2023-04-28

**Authors:** Skylar E. Davis, Anna K. Cook, Justin A. Hall, Yuliya Voskobiynyk, Nancy V. Carullo, Nicholas R. Boyle, Ahmad R. Hakim, Kristian M. Anderson, Kierra P. Hobdy, Derian A. Pugh, Charles F. Murchison, Laura J. McMeekin, Micah Simmons, Katherine A. Margolies, Rita M. Cowell, Alissa L. Nana, Salvatore Spina, Lea T. Grinberg, Bruce L. Miller, William W. Seeley, Andrew E. Arrant

**Affiliations:** 1 Department of Neurology, Center for Neurodegeneration and Experimental Therapeutics, Alzheimer’s Disease Center, Evelyn F. McKnight Brain Institute, University of Alabama at Birmingham, Birmingham, AL USA; 2 Department of Neurobiology, University of Alabama at Birmingham, Birmingham, AL USA; 3 Department of Biostatistics, University of Alabama at Birmingham, Birmingham, AL USA; 4 Department of Neuroscience, Southern Research, Birmingham, AL USA; 5 Department of Neurology, Memory and Aging Center, UCSF Weill Institute for Neurosciences, University of California, San Francisco, San Francisco, CA USA; 6 Department of Pathology, University of California, San Francisco, San Francisco, CA USA

**Keywords:** Frontotemporal dementia, Lysosome, Progranulin, TDP-43

## Abstract

Loss of function progranulin (*GRN*) mutations are a major autosomal dominant cause of frontotemporal dementia (FTD). Patients with FTD due to *GRN* mutations (FTD-*GRN*) develop frontotemporal lobar degeneration with TDP-43 pathology type A (FTLD-TDP type A) and exhibit elevated levels of lysosomal proteins and storage material in frontal cortex, perhaps indicating lysosomal dysfunction as a mechanism of disease. To investigate whether patients with sporadic FTLD exhibit similar signs of lysosomal dysfunction, we compared lysosomal protein levels, transcript levels, and storage material in patients with FTD-*GRN* or sporadic FTLD-TDP type A. We analyzed samples from frontal cortex, a degenerated brain region, and occipital cortex, a relatively spared brain region. In frontal cortex, patients with sporadic FTLD-TDP type A exhibited similar increases in lysosomal protein levels, transcript levels, and storage material as patients with FTD-*GRN*. In occipital cortex of both patient groups, most lysosomal measures did not differ from controls. Frontal cortex from a transgenic mouse model of TDP-opathy had similar increases in cathepsin D and lysosomal storage material, showing that TDP-opathy and neurodegeneration can drive these changes independently of progranulin. To investigate these changes in additional FTLD subtypes, we analyzed frontal cortical samples from patients with sporadic FTLD-TDP type C or Pick’s disease, an FTLD-tau subtype. All sporadic FTLD groups had similar increases in cathepsin D activity, lysosomal membrane proteins, and storage material as FTD-*GRN* patients. However, patients with FTLD-TDP type C or Pick’s disease did not have similar increases in lysosomal transcripts as patients with FTD-*GRN* or sporadic FTLD-TDP type A. Based on these data, accumulation of lysosomal proteins and storage material may be a common aspect of end-stage FTLD. However, the unique changes in gene expression in patients with FTD-*GRN* or sporadic FTLD-TDP type A may indicate distinct underlying lysosomal changes among FTLD subtypes.

**Supplementary Information:**

The online version contains supplementary material available at 10.1186/s40478-023-01571-4.

## Introduction

Loss-of-function mutations in progranulin (*GRN*) are a major autosomal dominant cause of frontotemporal dementia (FTD) [10, 29]. Most of these mutations cause progranulin haploinsufficiency [33, 36, 62, 79], which is thought to cause FTD in *GRN* mutation carriers (FTD-*GRN*). The presence of loss-of-function mutations on both *GRN* alleles, resulting in complete progranulin deficiency, causes the lysosomal storage disorder Neuronal Ceroid Lipofuscinosis (NCL) [2, 44, 82], showing that progranulin is necessary for maintaining lysosomal function.

Progranulin is a secreted pro-protein that is trafficked to lysosomes and cleaved into granulins [43, 106]. Progranulin and some granulins facilitate the activity of several lysosomal enzymes, including the protease cathepsin D (CatD) and enzymes involved in glycosphingolipid metabolism. Progranulin and some granulins interact with CatD and enhance its maturation and stability [13, 17, 18, 84, 104]. Progranulin also facilitates maturation of the glycosphingolipid-metabolizing enzymes β-hexosaminidase A (HexA) [26] and β-glucocerebrosidase (GCase) [8, 46, 85, 105] and regulates lysosomal levels of critical co-factors for glycosphingolipid metabolism, including prosaposin [106, 107] and BMP (bis(monoacylglycero)phosphate) [49, 55].

Brains of patients with FTD-*GRN* exhibit numerous lysosomal abnormalities. Cortical samples from patients with FTD-*GRN* have elevated levels of CatD and other lysosomal proteins [40], elevated levels of extracellular vesicles [4], and accumulation of lysosomal storage material [40, 90]. Cortical samples from patients with FTD-*GRN* also show signs of impaired glycosphingolipid metabolism such as reduced GCase activity [8, 85], low levels of BMP [14, 55], and accumulation of gangliosides [14]. These changes may be a sign of lysosomal dysfunction, which might drive FTD-*GRN* pathogenesis by impairing neuronal autophagy and promoting TDP-43 aggregation [24] or by shifting microglia to a reactive phenotype that destroys synapses and promotes TDP-43 mislocalization in neurons [39, 42, 56, 94, 100].

Lysosomal dysfunction may also be involved in pathogenesis of other genetic FTD subtypes [72, 86, 87]. FTD-causing mutations have been found in several genes involved in the autophagy-lysosomal or endolysosomal pathways, including *VCP* [92], *TBK1* [27, 35, 37, 67], *OPTN* [67], and *CHMP2B* [81]. Similar to patients with *GRN* mutations, brains of FTD patients with *CHMP2B* mutations contain higher levels of lysosomal storage material than controls [28]. *C9ORF72*, in which repeat expansions are the most common genetic cause of FTD and ALS, is also involved in aspects of lysosomal function [3, 42].

Given the evidence that lysosomal dysfunction may contribute to pathogenesis of several genetic FTD subtypes [72, 86, 87], it is possible that lysosomal dysfunction might contribute to pathogenesis of sporadic FTD, which comprises most FTD cases. To address this question, we investigated whether the lysosomal abnormalities of FTD-*GRN* patients [8, 40, 85, 90] are also present in patients with sporadic FTD. We began by comparing samples from patients with FTD-*GRN* or sporadic FTLD-TDP type A (frontotemporal lobar degeneration with TDP-43 pathology type A), the same type of TDP-43 pathology that occurs in patients with FTD-*GRN* [58]. To assess the relationship of these lysosomal abnormalities with TDP-43 pathology and neurodegeneration, we analyzed samples of frontal cortex, a degenerated brain region, and occipital cortex, a mostly spared brain region. We then extended this investigation to include a transgenic mouse model of TDP-opathy [93] and additional subtypes of sporadic FTLD-TDP (FTLD-TDP type C) and FTLD-tau (Pick’s disease).

## Materials and methods

### Patient brain samples

Post-mortem brain samples were provided by the Neurodegenerative Disease Brain Bank at the University of California, San Francisco. Brains were donated with the consent of the patients or their surrogates in accordance with the Declaration of Helsinki and the research was approved by the University of California, San Francisco Committee on Human Research. Tissue blocks were dissected from the orbital part of the inferior frontal gyrus and the inferior occipital cortex of 5 controls, 13 patients with FTD-*GRN*, and 7 patients each with sporadic FTLD-TDP type A, FTLD-TDP type C, or Pick’s disease. All patients with FTD-*GRN* carried a pathogenic variant in *GRN* and had FTLD-TDP type A identified at autopsy, except one (Table 1, case 11), who had a primary pathology diagnosis of Lewy body disease, with possible early FTLD-TDP type A pathology. Patients with sporadic FTLD-TDP Type A carried no pathogenic *GRN* variants, though one (Table 1, case 21) carried an intronic *GRN* variant of unknown significance. Patient characteristics are provided in Table 1. Clinical and neuropathological diagnoses were made using standard diagnostic criteria [38, 58, 59, 64, 69].

**Table 1:** Description of patients

#	Group	*GRN* Mutation*	Sex	Age at death	Clinical diagnosis**	Primary neuropath diagnosis***	PMI (h)
1	Ctrl	n/a	F	81	MCI, amnestic	Braak 2	30.3
2	Ctrl	n/a	M	77	MCI, executive	AGD	4.9
3	Ctrl	n/a	F	86	Control	CVD; AGD	7.8
4	Ctrl	n/a	M	76	Control	Braak 2, limbic AGD	8.2
5	Ctrl	n/a	F	86	Control	iLBD, brainstem predominant	6.4
6	GRN	c.1145del:p.Thr382Serfs*30	M	74	nfvPPA/CBS	FTLD-TDP-A	30.9
7^#^	GRN	c.347C > A:p.Ser116*	M	68	bvFTD	FTLD-TDP-A	13.5
8	GRN	c.264 + 2 T > C	F	73	nfvPPA/CBS	FTLD-TDP-A	20.7
9	GRN	c.1477C > T:p.Arg493*	F	66	bvFTD	FTLD-TDP-A	7.4
10	GRN	c.709-2A > G	M	64	bvFTD	FTLD-TDP-A	7.2
11	GRN	c.1256_1263dup:p.Ile422Glufs*72	M	66	DLB	LBD, FTLD-TDP-A	10.1
12	GRN	c.1A > T:p.Met1?	F	59	CBS	FTLD-TDP-A	9.5
13	GRN	c.1477C > T:p.Arg493*	F	70	PPA, unspecified	FTLD-TDP-A	9.1
14	GRN	c.1216C > T:p.Gln406*	F	56	bvFTD	FTLD-TDP-A	7.6
15	GRN	c.349 + 1G > A	F	78	mixed FTD	FTLD-TDP-A	19
16	GRN	c.328C > T:p.Arg110*	F	66	bvFTD	FTLD-TDP-A	17.1
17	GRN	c.708 + 1G > A	F	64	CBS	FTLD-TDP-A	10.5
18	GRN	c.1256_1263dup:p.Ile422Glufs*72	M	72	AD-type dementia	AD, FTLD-TDP-A	7.2
19	TDP-A	n/a	F	78	nfvPPA	FTLD-TDP-A	9
20	TDP-A	n/a	F	66	CBS	FTLD-TDP-A	17.3
21	TDP-A	n/a^†^	M	72	PPA-mixed	FTLD-TDP-A	23.8
22	TDP-A	n/a	M	63	CBS	FTLD-TDP-A	15.7
23	TDP-A	n/a	M	70	bvFTD	FTLD-TDP-A	8.5
24	TDP-A	n/a	F	73	bvFTD	FTLD-TDP-A	7.4
25	TDP-A	n/a	F	78	AD-type dementia vs. FTD	FTLD-TDP-A	8.1
26	TDP-C	n/a	F	69	svPPA	FTLD-TDP-C	10.7
27	TDP-C	n/a	M	66	svPPA	FTLD-TDP-C	10
28	TDP-C	n/a	F	68	svPPA	FTLD-TDP-C	8.4
29	TDP-C	n/a	M	66	svPPA	FTLD-TDP-C	14.5
30	TDP-C	n/a	M	75	svPPA	FTLD-TDP-C	3.8
31	TDP-C	n/a	F	75	svPPA	FTLD-TDP-C	7.9
32	TDP-C	n/a	F	71	svPPA	FTLD-TDP-C	13
33	Pick's	n/a	M	64	CBS	Pick's Disease	13.7
34	Pick's	n/a	F	78	nfvPPA	Pick's Disease	14.5
35	Pick's	n/a	F	67	AD-type dementia	Pick's Disease	6.8
36	Pick's	n/a	F	63	nfvPPA	Pick's Disease	4.3
37	Pick's	n/a	M	57	bvFTD	Pick's Disease	12.4
38	Pick's	n/a	F	73	nfvPPA	Pick's Disease	9.6
39	Pick's	n/a	M	78	svPPA	Pick's Disease	7.5

*All *GRN* mutations were heterozygous. **Disease considered most likely to explain the clinical syndrome. ***No control subject had limbic TDP-43 proteinopathy. ^#^case 7 was only analyzed for lysosomal storage markers in fixed tissue. ^†^case 21 was negative for pathogenic *GRN* mutations, but had intronic *GRN* variants of unknown significance. *PMI* postmortem interval, *AD* Alzheimer’s disease, *AGD* argyrophilic grain disease, *bvFTD* behavioral variant frontotemporal dementia, *CBS* corticobasal syndrome, *DLB* dementia with Lewy bodies, *LBD* Lewy body disease, *MCI* mild cognitive impairment, *nfvPPA* nonfluent variant primary progressive aphasia, *svPPA* semantic variant primary progressive aphasia

### Animals

Mice expressing wild-type human *TARDBP* under control of the *Thy-1* promoter (TDP + +) [93] were obtained from the Jackson Laboratory (#012,836). Mice used for this study were on a mixed genetic background, having been crossed from a C57Bl6J/SJL F1 background onto a C57Bl6/J background for 2–4 generations before use. Hemizygous transgenic mice were bred to produce nontransgenic and homozygous transgenic littermates for all experiments. Due to the severe motor deficits of homozygous transgenic mice of this line [93], mice were euthanized at 21 days of age for collection of brain samples. Both male and female mice were studied. Mice were housed in a facility accredited by the Association for Assessment and Accreditation of Laboratory Animal Care on a 12 h light/dark cycle with lights on at 6:00 AM. Mice had free access to food (Envigo #7917) and water throughout the study. All experiments were approved by the Institutional Animal Care and Use Committee of the University of Alabama at Birmingham.

### Tissue preparation

Prior to analysis by enzyme activity assays or immunoblot, tissue from frontal or occipital cortex of patients with FTD or frontal cortex of TDP-43 transgenic mice was homogenized in lysis buffer (50 mM Tris, 150 mM NaCl, 5 mM EDTA, 1% Triton X-100, 0.1% sodium deoxycholate) and centrifuged for 10 min at 5000 × *g*. For analysis of Triton-insoluble p-TDP-43, the resulting pellets were solubilized in 8 M urea. Protein concentration was determined by BCA assay (ThermoFisher) and uniform amounts of protein were used for subsequent activity assays and SDS-PAGE. Two samples were analyzed from each tissue block and averaged to give final results for each patient.

### Lysosomal enzyme activity assays

HexA and GCase activity were determined as previously described [8] using fluorogenic substrates (HexA-4-methylumbelliferyl-2-acetamido-2-deoxy-6-sulfate-β-D-glucopyranoside, Research Products International, GCase-4-Methylumbelliferyl β-D-glucopyranoside, MilliporeSigma) conjugated to the fluorophore 4-methylumbelliferone (4-MU). Fluorescence was measured using a Biotek Synergy LX plate reader and quantified relative to a standard curve of 4-MU run on each plate.

CatD activity was determined as previously described [7] using the fluorogenic cathepsin D/E substrate (7-methoxycoumarin-4-yl)acetyl-GKPILF ~ FRLK(2,4-dinitrophenyl)-D-R-NH_2_ (MilliporeSigma) [97]. Specific CatD activity was determined by subtracting fluorescence generated in the presence of the CatD inhibitor pepstatin A (Fisher Scientific). Lysates were incubated with substrate for 60 min at 37 °C prior to reading fluorescence on a Biotek Synergy LX plate reader. Fluorescence was normalized to control samples run on the same plate.

### Immunoblotting

Samples were subjected to SDS-PAGE on 10% TGX polyacrylamide gels (Bio-Rad) prior to transferring to Immobilon-FL PVDF membranes (MilliporeSigma). Membranes were blocked with protein-free blocking buffer (Thermo Scientific) before overnight incubation with primary antibody at 4 °C. The following day, membranes were incubated with species-matched IR-dye–conjugated secondary antibodies (Li-COR Biosciences) and scanned on an Odyssey scanner (Li-COR Biosciences). In some cases, blots were stripped (62.5 mM Tris, pH 6.7, 4% SDS, 85 mM β-mercaptoethanol) and re-probed for additional proteins. Bands were quantified using ImageStudio Lite software (Li-COR Biosciences), with the exception of low molecular weight GCase, which was quantified using ImageJ to enable clear separation from mature GCase.

### Immunostaining

Formalin-fixed blocks of the orbital part of the inferior frontal gyrus or inferior occipital cortex were provided by the Neurodegenerative Disease Brain Bank at the University of California, San Francisco, as described above. Brains from TDP-43 transgenic mice were prepared by transcardial perfusion with 0.9% saline, bisection into hemibrains, and 48-h post-fixation in 4% paraformaldehyde. All tissue was cryoprotected in 30% sucrose and cut into 30 µm sections on a sliding microtome (Leica).

Prior to immunostaining, sections from patient tissue were subjected to antigen retrieval in 10 mM sodium citrate, pH 6.0 at 80ºC for 2 h. Mouse brain sections did not require antigen retrieval. The sections were immunostained by overnight incubation with primary antibody, followed by species-matched secondary antibodies, as previously described [66]. Species-matched AlexaFluor-conjugated secondary antibodies (ThermoFisher) were used for immunofluorescence and autofluorescence was quenched using 1% Sudan Black B (Acros Organics). Biotinylated secondary antibodies (Vector Laboratories) and VectaStain Elite ABC reagent (Vector Laboratories) were used for chromogenic labeling with diaminobenzidene (MP Biomedicals).

### Antibodies

Antibodies used in this study are described in Table 2.

**Table 2:** Antibodies used for Immunoblotting and Immunostaining

Target	Application	Sample Type	Source/Cat. #	Dilution
CatD	IB	Patient	Santa Cruz Biotechnology, sc-	1:500
	IF		6486	1:500
CatD	IB	Mouse	R&D Systems, AF1029	1:500
	IF			1:1000
GCase	IB	Patient	MilliporeSigma, G4171	1:500
GCase	IF	Patient	R&D Systems, MAB7410	1:250
GFAP	IF	Patient/Mouse	Agilent, Z033429	1:1000
HexA	IB	Patient	Santa Cruz Biotechnology, sc-376777	1:500
HexA	IB	Mouse	Abcam, ab189865	1:500
Iba1	IF	Patient/Mouse	Wako, 019–19,741	1:500
LAMP-1	IB	Patient	Santa Cruz Biotechnology, sc-20011	1:1000
LAMP-1	IB	Mouse	Developmental Studies Hybridoma Bank, 1D4B	1:100
LAMP-2	IB	Patient	Santa Cruz Biotechnology, sc-18822	1:1000
MAP2	IF	Patient	ThermoFisher, PA1-10,005	1:1000
NeuN	IF	Patient	Abcam, ab177487	1:1000
NeuN	IF	Mouse	MilliporeSigma, Abn91	1:500
p-TDP-43 (Ser409/410)	IF	Mouse	Proteintech, 22,309–1-AP	1:1000
	IHC			1:60,000
	IB	Patient		1:500
SCMAS	IHC	Mouse	Abcam, ab181243	1:500

*CatD* cathepsin D, *GCase* β-glucocerebrosidase, *HexA* β-hexosaminidase A, *IB* immunoblot, *IF* immunofluorescence, *IHC* immunohistochemistry, *SCMAS* subunit C of mitochondrial ATP synthase

### Autofluorescence

Brain sections from frontal and occipital cortex were washed with PBS, mounted onto Colorfrost Plus slides (Fisher Scientific), and coverslipped with Vectashield HardSet mounting medium containing DAPI (Vector Laboratories). Colocalization of autofluorescence with cell type markers was assessed by immunostaining for MAP2, Iba1, or GFAP as described above, but without quenching autofluorescence with Sudan Black B dye.

### Sudan black B staining

Brain sections were mounted onto Colorfrost Plus slides (Fisher Scientific) and dried overnight prior to staining. Slides were then washed twice with water, immersed in 70% ethanol for one minute, then stained for 20 min in 0.1% Sudan Black B (Acros Organics) in 70% ethanol. Slides were then washed twice each with PBS and water before coverslipping with Vectashield HardSet mounting medium (Vector Laboratories).

### Imaging and analysis

Patient brain sections processed for autofluorescence and Sudan Black B were imaged at 20 × on a Nikon upright microscope or an EVOS M5000 imaging system (ThermoFisher). Images were taken of cortical layer II/III from 2–3 sections from each patient and averaged to give final values. The number of autofluorescent or Sudan Black B-positive particles per field of view was determined using ImageJ. Due to variability in background fluorescence and staining, the Triangle autothreshold function [99] was used to detect autofluorescence and the Yen autothreshold function [98] was used to detect Sudan Black B. Preliminary trials with these functions produced very similar thresholds as investigators blinded to sample identity.

Images of p-TDP-43, autofluorescence, and SCMAS from mouse brain sections were taken at 20X on an EVOS M5000 imaging system (ThermoFisher). Staining was quantified with ImageJ by applying a uniform threshold to all images. Immunofluorescent images for colocalization were obtained at 20X on a Nikon Ti2-C2 confocal microscope.

### Nanostring analysis

A custom Nanostring nCounter panel was designed to assess expression of 42 endolysosomal genes, with normalization to the housekeeping genes *AARS*, *CCDC12*, *FAM104A*, *HPRT1*, and *PGK1*. RNA was extracted from samples of frontal and occipital cortex using the Qiagen RNEasy kit, then 200 ng of RNA from each sample was analyzed using an nCounter analysis system (Nanostring) and nSolver 4.0 software (Nanostring) in UAB’s Nanostring laboratory.

### qPCR

For mouse brain samples, quantitative reverse-transcription PCR was performed as previously described [34]. RNA was extracted from frontal cortex of nontransgenic and TDP++ littermates with Trizol (ThermoFisher). RNA samples were treated with DNase (Promega), then reverse transcribed with the High Capacity cDNA Reverse Transcription Kit (ThermoFisher). qPCR was conducted using Taqman assays (ThermoFisher) for *Hexa* (Mm00599877_m1), *Ctsd* (Mm00515586_m1), *Gusb* (Mm01197698_m1), *Atp6v1a* (Mm01343719_m1), *Atp6v0a2* (Mm00441838_m1), *Lamp1* (Mm00495262_m1), *Cd63* (Mm01966817_g1), *Cd9* (Mm00514275_g1), and *Grn* (Mm01245914_g1) using JumpStart Taq Readymix (MilliporeSigma), and normalized to expression of *Actb* (Mm00607939_s1).

For patient samples, RNA was extracted with the Qiagen RNEasy kit, treated with DNase (ThermoFisher), and reverse transcribed using Biorad iScript. qPCR was conducted using pre-designed Prime-Time qPCR primers (Integrated DNA Technologies) and Power SYBR Green master mix (ThermoFisher). The following primer sets were used: *HEXA* (Hs.PT.58.24457208), *CTSD* (Hs.PT.58.27568031), *GUSB* (Hs.PT.58v.27737538), *IDUA* (Hs.PT.58.40058589), *LAMP1* (Hs.PT.58.27192505), *CD63* (Hs.PT.58.25219306), *GRN* (Hs.PT.58.2528960.g), and *PSAP* (Hs.PT.58.744083), and normalized to expression of *HPRT1* (Hs.PT.58v.45621572). Two samples were analyzed from each tissue block and averaged to give final results for each patient.

### Small molecule fluorescent in situ hybridization (FISH)

Small molecule fluorescent in situ hybridization (FISH, RNAscope) was performed to localize and quantify transcript for *Ctsd* (cat #520,571, Advanced Diagnostics/ACD) in mouse brain tissue. Brains from non-transgenic or TDP++ mice were fresh frozen on dry ice, sectioned at 20 µm on a cryostat, and re-frozen at -80° C until use. FISH was performed according to manufacturer’s instructions and as previously described [34], using four mice/group, four coronal hemisections/mouse. Cortical glutamatergic neurons and parvalbumin-expressing interneurons were identified using probes for *Slc17a7* (cat #416,631-C2, Advanced Diagnostics) and *Pvalb* (cat #421,931-C3, Advanced Diagnostics), respectively, using the RNAscope Mulitplex Fluorescent Assay V1 (Advanced Diagnostics). Images were collected on a Nikon A1 + confocal microscope and exported as tiffs into ImageJ for analysis [34, 76, 80]. Thresholds were set using samples from non-transgenic mice, regions of interest were selected by *Slc17a7* or *Pvalb*-positivity in the same sections, and *Ctsd* was quantified by region-of-interest, taking into consideration the area of the cell (generating a mean pixel density value).

### Electron microscopy

Frontal cortices were rapidly dissected after euthanizing mice, then fixed overnight at 4 °C in 6% glutaraldehyde and 2% paraformaldehyde in 0.15 M cacodylate buffer with 1 mM Ca^++^ and 2 mM Mg^++^. The following day, the tissue was rinsed three times in 0.15 M cacodylate buffer, then post-fixed for 90 min at room temperature in 1% osmium tetroxide in 0.15 M cacodylate buffer. The tissue was then rinsed three more times and processed through a graded ethanol series followed by three changes in propylene oxide. The tissue was infiltrated overnight with a 1:1 solution of propylene oxide and Epon-812 resin, followed by three incubations in 100% resin for two hours each. Tissue pieces were arranged in embedding molds, embedded in fresh resin, and polymerized at 65 °C overnight. The samples were then sliced, mounted onto copper grids, and imaged on a Technai Spirit T12 transmission electron microscope (ThermoFisher).

### Statistics

Most data are shown in box and whisker plots with the box drawn from the 25^th^ to 75^th^ percentiles, a line at the median value, and whiskers extending to the minimum and maximum data points. Tables containing all patient data are available in Additional File 2. For all analyses, two-tailed *p* values were calculated with α set at 0.05. Data were tested for unequal variance using Bartlett’s test or F test, and for non-normal distribution using the D’Agostino-Pearson test in GraphPad Prism 9. Data that failed to meet assumptions of equal variance or normality were either log-transformed or analyzed by nonparametric tests. Except where noted, data were analyzed with GraphPad Prism 9.

Enzyme activity, protein levels, Triton-insoluble p-TDP-43, and lysosomal storage material in patient samples were analyzed by one-way ANOVA with a factor of patient group. Significant group effects were followed by Fisher’s LSD post-hoc test. Due to unequal variance, levels of low molecular weight GCase, Triton-insoluble p-TDP-43, and lysosomal storage material in patients were log-transformed prior to analysis. The relationship between p-TDP-43 and lysosomal protein levels was assessed using Spearman correlation. Mouse enzyme activity and protein levels were analyzed by *t* test, and *Ctsd *in situ hybridization was analyzed with Mann–Whitney test (mean values per mouse) or Kolmogorov–Smirnov test (cumulative frequency of *Ctsd* labeling). Lysosomal storage material in mice was analyzed with Mann–Whitney test. Due to analysis of multiple genes that were expected to exhibit similar changes relative to controls, qPCR data from both patient and mouse samples were analyzed by MANOVA using IBM SPSS Statistics 27. Group significance for individual genes was determined by between-subjects test. For patient qPCR data, this was followed with pair-wise comparisons between patient groups using Tukey’s post-hoc test. Due to unequal variance, both patient and mouse qPCR data were log-transformed prior to analysis.

Analyses of Nanostring data were conducted with R 4.0 with additional utility from the Bioconductor suite of packages, with specific application of the Limma, Glimma, and DeSeq2 packages. Nanostring data were analyzed with general linear mixed-effects models to evaluate the impact of patient group on differential expression of mRNA. Abundance of mRNA profiles assumed a Gamma distribution using a natural log link function to account for the right-skewed scale distribution. Once samples were normalized to adjust for batch effects, GLME models were applied to each of transcripts of interest with fixed effects covariates for brain region (occipital vs orbital), patient group (control vs FTD-*GRN* vs sporadic FTLD-TDP type A) and their interaction. Random effects blocked on individual patients who provided tissue from both brain regions. Specific contrasts of interest identified group effects between control patients and the two groups of patients with FTLD while controlling for brain region effects. For significance of differential expression between patients with FTD-*GRN* or sporadic FTLD-TDP type A versus controls, changes in mRNA expression for patients required a minimum 50% fold change in either direction relative to controls to be considered clinically meaningful, with statistical significance for *p*-values less than 0.05 after False Discovery Rate correction of 5% to account for multiple comparisons.

## Results

### Similar increases in lysosomal proteins in frontal cortex of patients with FTD-GRN and sporadic FTLD-TDP type A

Prior reports show that frontal cortex from patients with FTD-*GRN* has elevated levels of CatD, HexA, and other lysosomal proteins [8, 40], as well as reduced levels of mature GCase and accumulation of incompletely glycosylated GCase [8, 85]. We therefore analyzed frontal cortex from controls, patients with FTD-*GRN*, and patients with sporadic FTLD-TDP type A for CatD, HexA, and GCase enzyme activity and protein levels.

**Fig. 1:**
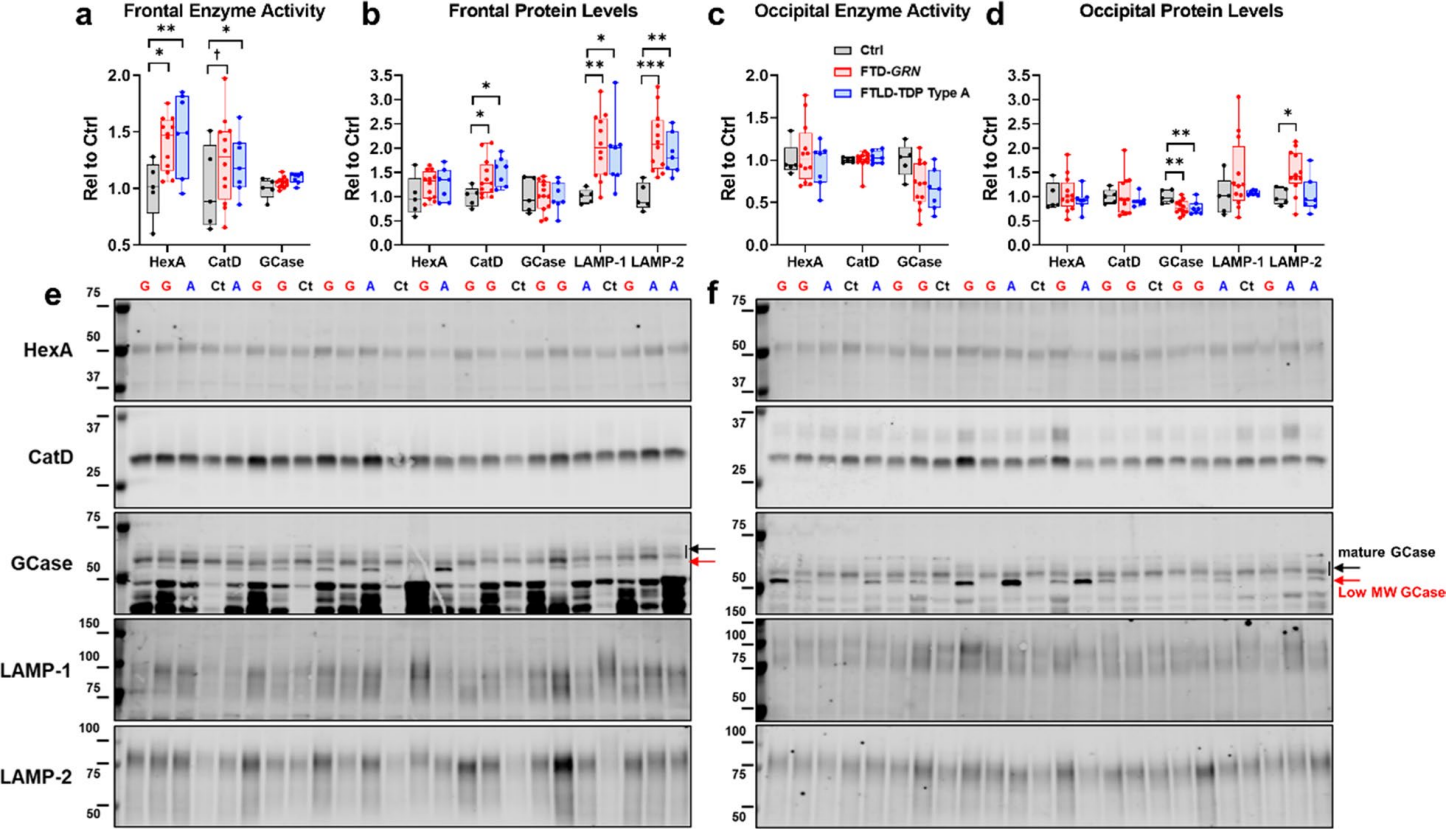
Increases in lysosomal enzyme activity and protein levels in frontal cortex of patients with FTD-*GRN* and sporadic FTLD-TDP type A. **a** Analysis of lysates from frontal cortex of patients with FTD-*GRN* or sporadic FTLD-TDP type A revealed similar increases in HexA activity (ANOVA, *p* = 0.0171) and CatD activity (ANOVA, *p* = 0.0404) in each patient group. No changes in GCase activity were detected (ANOVA, *p* = 0.4053). **b** Lysosomal protein levels followed a similar pattern, with both patient groups exhibiting increased levels of mature CatD (ANOVA, *p* = 0.0429), LAMP-1 (ANOVA, *p* = 0.0170), and LAMP-2 (ANOVA, *p* = 0.0019). **c** In contrast, no significant changes in enzyme activity were detected in occipital cortex, though there was a trend for reduced GCase activity in FTD patients (ANOVA, *p* = 0.0595). **d** Levels of mature GCase protein were reduced in occipital cortex of both patient groups (ANOVA, *p* = 0.0159) and the only elevated lysosomal protein in occipital cortex was LAMP-2, which was only elevated in patients with FTD-*GRN* (ANOVA, *p* = 0.0232). Immunoblots for frontal cortex are shown in **e** and for occipital cortex are shown in **f**. HexA = β-hexosaminidase A, CatD = cathepsin D, GCase = β-glucocerebrosidase. In **e**, **f** Ct = control, G = FTD-*GRN*, and A = sporadic FTLD-TDP type A. Molecular weight markers are identified by weight in kDa for each blot. ^†^*p* < 0.1, *p < 0.05, **p < 0.01, and ***p < 0.001 by Fisher’s LSD post-hoc test. n = 5 controls, 12 patients with FTD-*GRN*, and 7 patients with sporadic FTLD-TDP type A

Across all measures, patients with FTD-*GRN* and sporadic FTLD-TDP type A exhibited similar changes relative to controls. Both patient groups exhibited elevated HexA activity (Fig. 1a), as well as elevated levels of mature CatD and the lysosomal membrane proteins LAMP-1 and LAMP-2 (Fig. 1b). In contrast to prior observations [8, 85], neither group exhibited a deficit in GCase activity (Fig. 1a) or mature GCase protein levels (Fig. 1b). Most patients from both FTLD groups exhibited a low molecular weight GCase band that we previously found to be composed of incompletely glycosylated GCase (Fig. 1e) [8]. This band was very faint in all but one control. Levels of this low molecular weight GCase were significantly elevated versus controls in patients with sporadic FTLD-TDP type A (Additional file 1: Fig. S1), with a similar trend in patients with FTD-*GRN.*

### Limited changes in lysosomal proteins in occipital cortex of patients with FTD-GRN and sporadic FTLD-TDP type A

To determine whether these lysosomal changes were limited to a degenerated brain region, we measured the same lysosomal markers in occipital cortex, a region that was relatively spared from FTLD-TDP pathology.

In occipital cortex, neither patient group exhibited changes in HexA or CatD (Fig. 1c,d), but there was a trend for reduced GCase activity (Fig. 1c, ANOVA *p* = 0.0595) in both FTLD patient groups. Levels of mature GCase were significantly reduced in both FTLD patient groups (Fig. 1d), many of whom exhibited some accumulation of low molecular weight GCase (Fig. 1e), though levels of low molecular weight GCase did not statistically differ from controls (Additional file 1: Fig. S1). LAMP-1 levels were not significantly elevated in either FTLD patient group, but LAMP-2 levels were elevated in patients with FTD-*GRN* (Fig. 1d). In summary, the increases in lysosomal activity and protein levels were largely limited to frontal cortex in both FTLD patient groups.

### Association of increased CatD and LAMP-1 with accumulation of p-TDP-43 in frontal cortex

The more dramatic increase in lysosomal proteins in frontal cortex than in occipital cortex of patients with FTD-*GRN*, as well as the presence of similar increases in patients with sporadic FTLD-TDP type A, suggested that these changes may be more associated with aspects of FTLD-TDP type A pathology such as TDP-opathy, neurodegeneration, or neuroinflammation than with progranulin insufficiency. To gain insight into the relationship of TDP-opathy with increases in lysosomal proteins, we conducted immunoblots for p-TDP-43 (Ser409/410) on the Triton-insoluble pellets from lysates used to determine enzyme activity and protein levels. These immunoblots revealed a roughly 25 kDa p-TDP-43 band that was detectable only in patients with FTD-*GRN* or sporadic FTLD-TDP type A (Additional file 1: Fig. S2a,b). Both patient groups exhibited similar levels of 25 kDa p-TDP-43 on average, so we analyzed the correlation across FTLD groups of 25 kDa p-TDP-43 with levels of proteins that were significantly elevated in patients with FTLD: CatD, LAMP-1, LAMP-2, and low molecular weight GCase (Additional file 1: Fig. S2c–f). These analyses revealed a significant correlation of 25 kDa p-TDP-43 with CatD and LAMP-1, with a similar trend for LAMP-2. Levels of low molecular weight GCase were not correlated with levels of 25 kDa p-TDP-43.

### Cellular distribution of altered lysosomal proteins in frontal cortex of patients with FTD-GRN and sporadic FTLD-TDP type A

Since neuroinflammation could be an important factor driving the increase in lysosomal proteins in the frontal cortex, we conducted co-immunotaining to investigate whether these proteins were highly expressed in glia. We immunostained for progranulin, CatD, and GCase with markers of neurons (NeuN), microglia (Iba1), and astrocytes (GFAP) in frontal cortex of controls and patients with FTD-*GRN* or sporadic FTLD-TDP type A. Consistent with a prior report [25], we observed strong progranulin immunoreactivity in microglia (Additional file 1: Fig. S3a), suggesting that high progranulin expression by reactive microglia may mask the progranulin haploinsufficiency of patients with *GRN* mutations.

In contrast, we observed robust CatD immunoreactivity in neurons from both patient groups (Additional file 1: Fig. S3b), as well as some labeling in both microglia and astrocytes. Based on this labeling pattern, the increased CatD activity in FTLD patient tissue could be driven by changes in both neurons and glia.

Consistent with our prior observations [8], we observed lower GCase immunoreactivity in neurons from frontal cortex of patients with FTD-*GRN* (Additional file 1: Fig. S3c), but also observed some GCase immunoreactivity in astrocytes. Astrogliosis might therefore explain why we did not observe lower mature GCase levels in lysates of frontal cortex of patients with FTD-*GRN*, but did observe lower GCase levels in occipital cortex, which should have less neuroinflammation in these patients.

### Similar increases in lysosomal transcript levels in frontal cortex of patients with FTD-GRN and sporadic FTLD-TDP type A

**Fig. 2:**
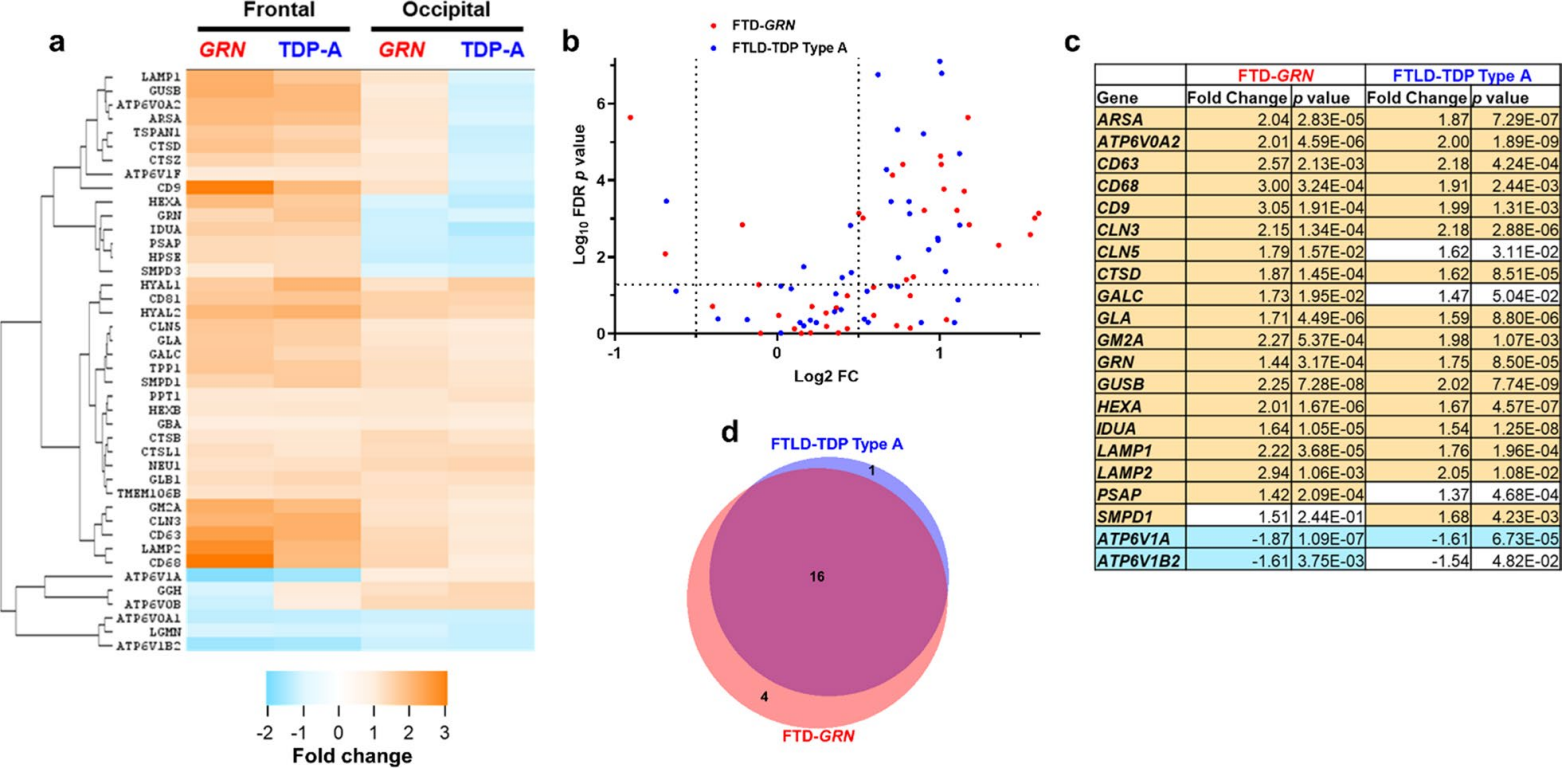
Similar changes in lysosomal gene expression in patients with FTD-*GRN* and sporadic FTLD-TDP type A. **a** Nanostring analysis of 42 genes related to endolysosomal function revealed similar changes in gene expression in frontal cortex of patients with FTD-*GRN* and patients with sporadic FTLD-TDP type A. **b**, **c** Statistical analysis revealed 20 differentially expressed genes versus control in frontal cortex across both patient groups, with no significant changes versus control in occipital cortex. **c**, **d** There was strong overlap of differentially expressed genes across both patient groups, with 16/20 genes exhibiting altered expression in both groups. Shaded cells in **c** indicate statistical significance after correction for multiple comparisons. n = 5 controls, 12 patients with FTD-*GRN*, and 7 patients with sporadic FTLD-TDP type A. In **a** *GRN* = FTD-*GRN* and TDP-A = FTLD-TDP type A

Having seen similar region-dependent changes in a small set of lysosomal proteins among patients with FTD-*GRN* and sporadic FTLD-TDP type A, we analyzed the expression of 42 lysosomal genes using a Nanostring panel to gain a broader view of lysosomal changes in these patients. This panel consisted of transcripts for late endosomal and lysosomal membrane proteins, enzymes, and ion channels (Fig. 2a). In frontal cortex, the majority of genes on the panel trended toward increased expression relative to controls (Fig. 2a), with a total of 21 genes reaching statistical significance (Fig. 2b,c). Of these 21 genes, 16 were shared between patients with FTD-*GRN* and sporadic FTLD-TDP type A (Fig. 2d). Consistent with enzyme activity and immunoblots, transcripts for *HEXA*, *CTSD*, *LAMP1*, and *LAMP2* were more abundant in each patient group relative to controls (Fig. 2c). *GRN* was also elevated in both patient groups, consistent with a prior study of patients with FTD-*GRN* [25]. Two genes encoding vacuolar ATPase subunits were downregulated, one of which was shared between patients with FTD-*GRN* and FTLD-TDP type A (Fig. 2b,c). In occipital cortex, no changes in gene expression reached statistical significance (Fig. 2a).

### Patients with FTD-GRN and sporadic FTLD-TDP type A exhibit signs of lysosomal storage material in frontal cortex

**Fig. 3:**
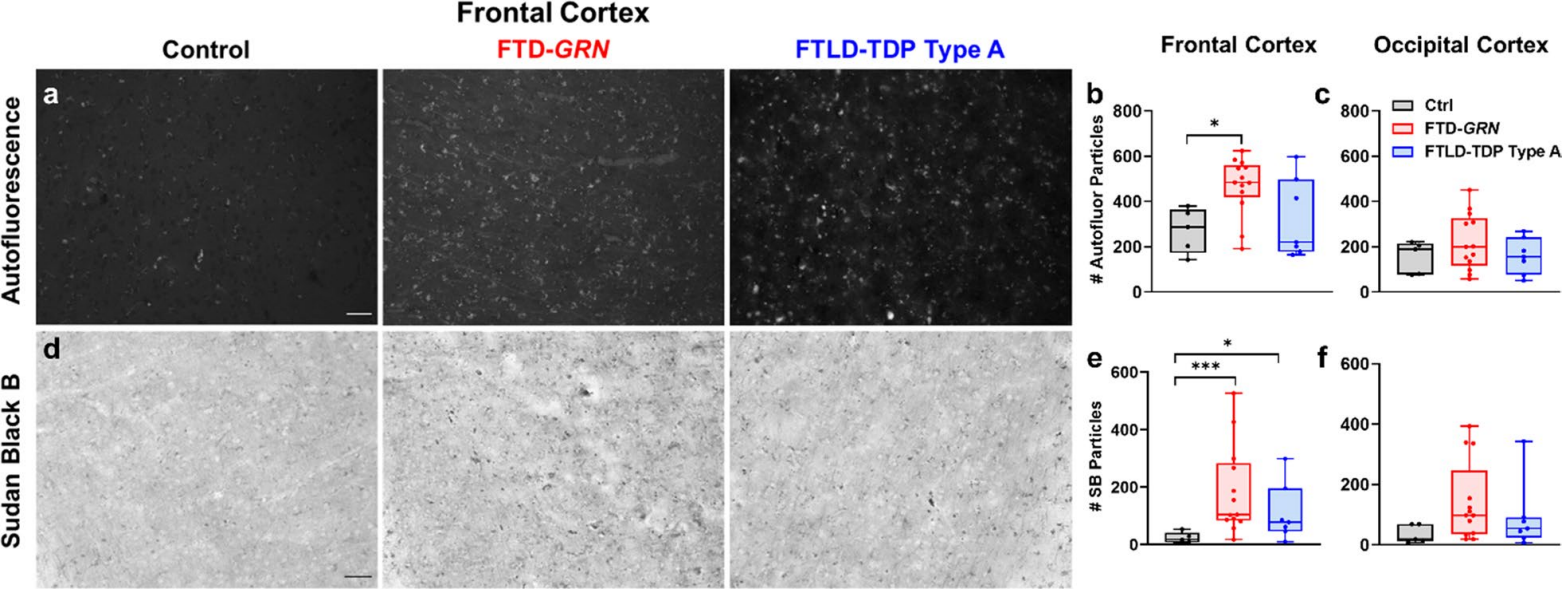
Signs of lysosomal storage material in frontal cortex of patients with FTD-*GRN* or sporadic FTLD-TDP type A. Patients with FTD-*GRN* exhibited a greater number of autofluorescent particles than controls in frontal cortex (ANOVA effect of group, *p* = 0.0243), while patients with sporadic FTLD-TDP type A did not differ from controls. **c** No difference was detected in autofluorescent particles in the occipital cortex of either group versus control (ANOVA effect of group, *p* = 0.4988). **d**, **e** Patients with FTD-*GRN* also exhibited a greater number of Sudan Black B-positive particles than controls in frontal cortex (ANOVA effect of group, *p* = 0.0022), as did patients with sporadic FTLD-TDP type A. **f** Levels of Sudan Black B-positive particles in occipital cortex of patients with FTD-*GRN* or FTLD-TDP type A did not significantly differ from controls (ANOVA effect of group, *p* = 0.0819). Scale bars in **a**, **d** represent 50 μm. *p < 0.05 and ***p < 0.001 by Fisher’s LSD post-hoc test. n = 5 controls, 13 patients with FTD-*GRN*, and 7 patients with sporadic FTLD-TDP type A

We next analyzed accumulation of lysosomal storage material by measuring autofluorescence and staining with Sudan Black B. Consistent with a prior report [90], patients with FTD-*GRN* had more autofluorescent (Fig. 3a,b) and Sudan Black B-positive particles (Fig. 3d,e) in frontal cortex than controls. Patients with sporadic FTLD-TDP-A had more Sudan Black B-positive particles in frontal cortex than controls (Fig. 3d,e), but did not have more autofluorescent particles. Neither FTLD patient group had significantly elevated autofluorescence (Fig. 3c) or Sudan Black B labeling (Fig. 3f) in occipital cortex, though patients with FTD-*GRN* had a trend for increased levels of Sudan Black B-positive particles (ANOVA, *p* = 0.0819).

### A mouse model of TDP-opathy replicates the increased CatD expression and accumulation of lysosomal storage material observed in FTLD-TDP type A patients

Since patients with FTD-*GRN* and sporadic FTLD-TDP type A had similar lysosomal abnormalities that were generally limited to the degenerated frontal cortex and some of which were associated with levels of p-TDP-43 (Additional file 1: Fig. S2), we hypothesized that TDP-opathy, neurodegeneration, and/or neuroinflammation might be sufficient to drive these lysosomal abnormalities. To test this hypothesis, we analyzed lysosomal phenotypes in a transgenic mouse model of TDP-opathy that expresses wild-type human TDP-43 under control of the *Thy1* promoter [93]. When bred to homozygosity (TDP++ mice), these mice exhibit TDP-43 aggregation (Additional file 1: Fig. S4), neuronal loss, and gliosis [93] by 21 days of age.

**Fig. 4:**
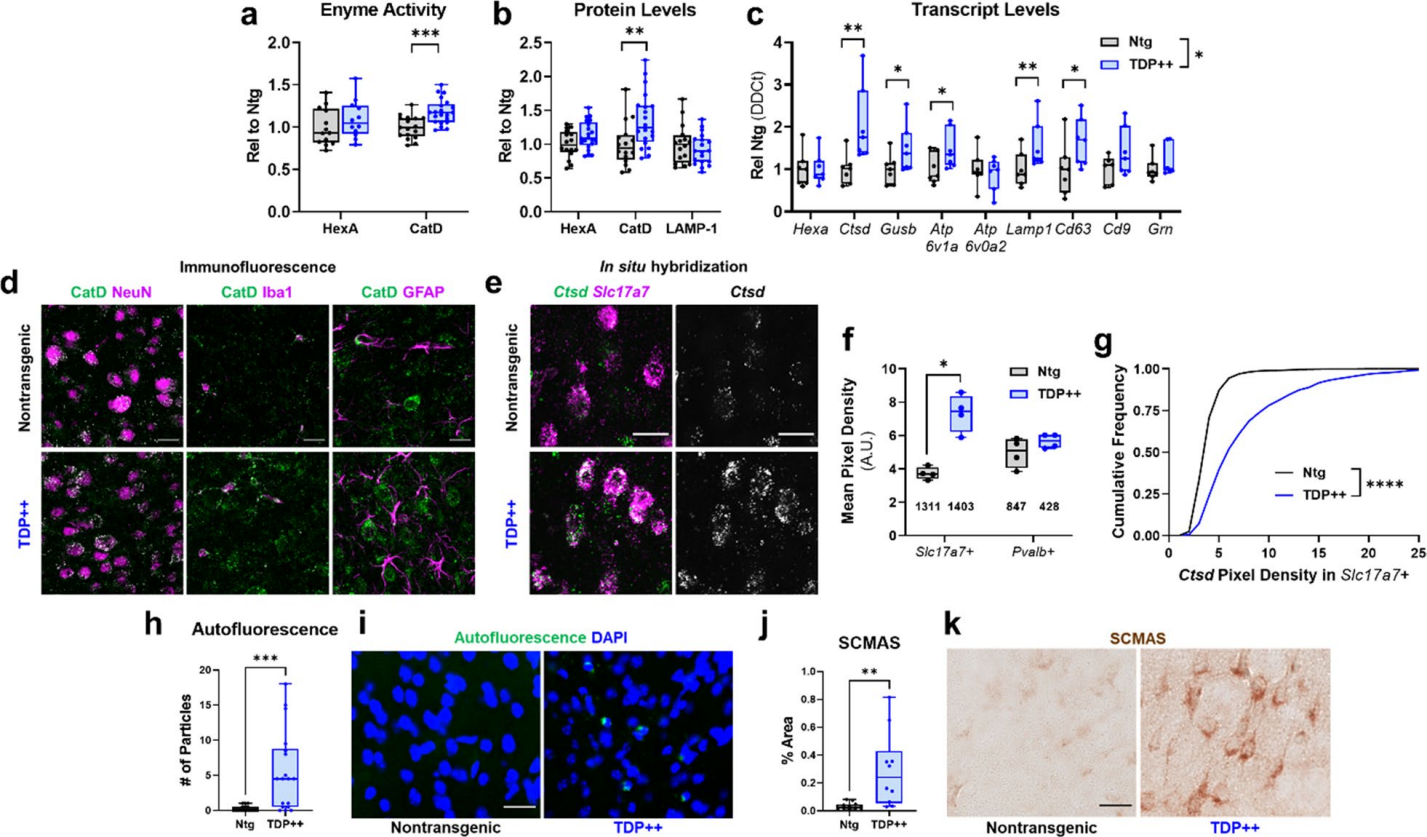
A mouse model of TDP-opathy partially replicates the lysosomal abnormalities observed in patients with FTD-*GRN* or sporadic FTLD-TDP type A.** a** Homozygous TDP-43 transgenic mice (TDP++) had elevated CatD activity versus nontransgenic littermates (*t* test, *p* = 0.0004, n = 17–21 mice per genotype), though HexA activity did not differ between groups. **b** TDP++ mice had a corresponding increase in mature CatD levels (*t* test, *p* = 0.0075, n = 16–21 mice per genotype), but no significant changes in HexA (*t* test, *p* = 0.0704) or LAMP-1 (*t* test, *p* = 0.3807). **c** TDP++ mice also exhibited increases in several lysosomal genes that were elevated in patients with FTLD (MANOVA effect of genotype, *p* = 0.019, n = 7 mice per genotype). **d** CatD immunoreactivity in TDP++ mice was mostly neuronal, but also present in glia. **e**–**g**, in situ hybridization for *Ctsd* confirmed an increase in *Ctsd* expression in cortical vGlut1-positive (*Slc17a7*+) excitatory neurons (Mann–Whitney test, *p* = 0.0286), but not in parvalbumin-positive interneurons (Mann–Whitney test, *p* = 0.3429, n = 4 mice per genotype, number of cells analyzed is noted in **f**). The cumulative frequency of *Ctsd* labeling in *Slc17a7* + excitatory neurons is shown in **g** (Kolmogorov–Smirnov test, *p* < 0.0001). TDP++ mice also accumulated storage material in frontal cortex, with an increase in autofluorescent particles (**h**, **i** Mann–Whitney test, *p* = 0.0002, n = 16–18 mice per genotype) and SCMAS (**j**, **k** Mann–Whitney, *p* = 0.0015, n = 10–11 mice per genotype). Scale bars represent 20 μm in (**d**, **e**, **i**, **k**). *p < 0.05, **p < 0.01, and ***p < 0.001 by *t* test in **a** and **b**, MANOVA between-subjects test in (**c**), ANOVA with Fisher’s LSD post-hoc test in (**f**), Kolmogorov–Smirnov test in (**g**), and Mann–Whitney test in (**h**, **j**)

Analysis of frontal cortex from 21 day-old TDP++ mice revealed increases in CatD activity and mature CatD levels similar to those observed in FTLD patients (Fig. 4a,b), though HexA and LAMP-1 levels were not significantly altered. Analysis of a subset of genes with differential expression in FTLD patients (Fig. 2) revealed that TDP++ mice replicated the increases in *Ctsd*, *Gusb*, *Lamp1*, and *Cd63* (Fig. 4c), though the mice exhibited an opposite change in *Atp6v0a2* from that observed in patients. As in FTD patients, CatD protein exhibited a heavily neuronal localization (Fig. 4d), and in situ hybridization revealed an increase in *Ctsd* mRNA expression in excitatory (*Slc17a7* +) neurons with no change in expression in local fast-spiking (*Pvalb* +) interneurons (Fig. 4e–g).

We next examined frontal cortex of 21 day-old TDP++ mice for lysosomal storage material by assessing levels of autofluorescence and subunit C of mitochondrial ATP synthase (SCMAS). SCMAS is a marker of lipofuscin [30, 41, 50] that is also elevated in *Grn*^*–/–*^ mice [7, 40], which model the lipofuscinosis of homozygous *GRN* carriers [2, 44, 82]. TDP++ mice had higher levels of both autofluorescence (Fig. 4h,i) and SCMAS (Fig. 4j,k) in frontal cortex than nontransgenic littermates, indicating accumulation of lysosomal storage material. Notably, the autofluorescent material was less abundant than observed in patients, perhaps due to the young age of these mice. The autofluorescent material was present in both neurons and microglia, and exhibited a punctate appearance rather than the granular morphology typical of lipofuscin (Additional file 1: Fig. S5). However, electron microscopy revealed deposits with similar appearance as storage material from *Grn*^*–/–*^ mice (Additional file 1: Fig. S5).

In summary, TDP++ mice partially replicated the lysosomal changes of patients with FTD-*GRN* or sporadic FTLD-TDP type A, particularly the increases in *Ctsd* transcript, CatD protein/activity, and potential lysosomal storage material. With the caveat that these data were obtained from young mice that overexpress TDP-43, these findings suggest that TDP-opathy, neurodegeneration, and/or neuroinflammation can drive these changes independently of progranulin haploinsufficiency.

### Patients with multiple sporadic FTLD subtypes exhibit similar lysosomal abnormalities as patients with FTD-GRN

To determine whether the lysosomal changes observed in patients with FTD-*GRN* or sporadic FTLD-TDP type A are specifically associated with TDP-opathy or may be more generally associated with neurodegeneration or neuroinflammation, we extended our investigation to include patients with a subtype of FTLD-TDP not associated with *GRN* mutations (type C) or a subtype of FTLD-tau (Pick’s disease). For this analysis, new slices were collected from the previously used tissue blocks of frontal cortex from controls and patients with FTD-*GRN* or sporadic FTLD-TDP type A and analyzed in parallel with slices from tissue blocks of frontal cortex from patients with FTLD-TDP type C or Pick’s disease.

**Fig. 5:**
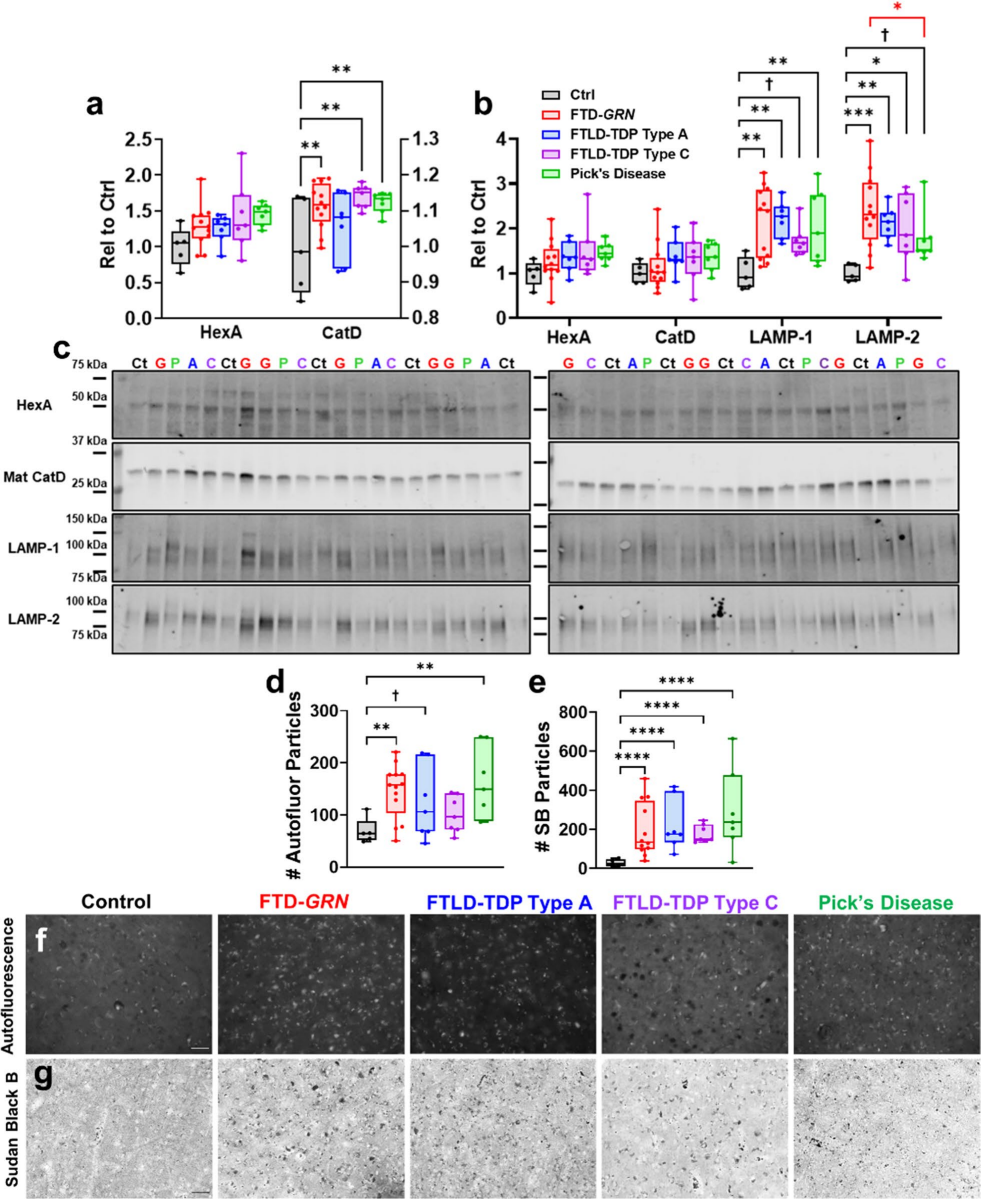
Similar lysosomal protein changes and storage material accumulation in frontal cortex of patients with multiple FTLD subtypes. Frontal cortex from patients with FTD-*GRN*, FTLD-TDP type C, or Pick’s Disease exhibited increased CatD activity versus controls (**a**, ANOVA effect of group, *p* = 0.0109). HexA activity was not significantly different from controls (ANOVA effect of group, *p* = 0.1079), though all FTLD groups exhibited trends for increased activity. **b**, **c** All FTLD patient groups also exhibited at least trends for elevated levels of LAMP-1 (ANOVA effect of group, *p* = 0.013) and LAMP-2 (ANOVA effect of group, *p* = 0.0059). **d**, **e** Patients with FTD-*GRN* and Pick’s disease exhibited higher numbers of autofluorescent particles compared to controls, and patients with FTLD-TDP type A exhibited a similar trend (ANOVA effect of group, *p* = 0.0254). **f**, **g** All FTLD patient groups had higher numbers of Sudan Black B-positive particles than controls (ANOVA effect of group, *p* < 0.0001). In **a** HexA data are scaled to the left y-axis and CatD data are scaled to the right y-axis. ^†^*p* < 0.1, *p < 0.05, **p < 0.01, ***p < 0.001, and ****p < 0.0001 by Fisher’s LSD post-hoc test. Black lines and symbols indicate difference from controls. The red line and symbol in **b** indicate difference from FTD-*GRN*. n = 5 controls, 12–13 patients with FTD-*GRN*, 7 patients with FTLD-TDP type A, 7 patients with FTLD-TDP type C, and 7 patients with Pick’s disease. Abbreviations for **c** are: Ct = control, G = FTD-*GRN*, A = FTLD-TDP type A, C = FTLD-TDP type C, P = Pick’s disease. Scale bars in **f** and **g** represent 50 μm

Analysis of lysosomal enzyme activity revealed a non-significant trend for increased HexA activity (Fig. 5a, ANOVA effect of patient group, *p* = 0.1079) among all FTLD patient groups. CatD activity was significantly increased in patients with FTD-*GRN*, FTLD-TDP type C, and Pick’s disease. Similarly, all FTLD patient groups exhibited increases in LAMP-1 and/or LAMP-2 (Fig. 5b) relative to controls. We did not observe significant increases in HexA activity or CatD protein levels versus controls as in Fig. 1, perhaps due to loss of statistical power with the increased number of comparisons.

As in Fig. 1, levels of mature GCase did not differ between controls and patients with FTLD, but patients with FTD-*GRN* or FTLD-TDP type A exhibited significant increases in low molecular weight GCase (Additional file 1: Fig. S6). Patients with FTLD-TDP type C did not accumulate low molecular weight GCase, and had significantly lower levels than patients with FTD-*GRN*. However, patients with Pick’s disease had intermediate levels of low molecular weight GCase that did not statistically differ from either controls or patients with FTD-*GRN*.

Analysis of lysosomal storage material revealed an increased number of autofluorescent particles (Fig. 5d,e) in patients with FTD-*GRN* and Pick’s disease, with a similar trend for patients with FTLD-TDP type A. All FTLD patient groups exhibited an increase in Sudan Black B-positive particles relative to controls (Fig. 5f,g). Fluorescent immunostaining revealed that neurons and astrocytes accumulated autofluorescent storage material, with microglia exhibiting some storage material as well (Additional file 1: Fig. S7).

In summary, all groups of patients with sporadic FTLD exhibited generally similar changes in lysosomal proteins as patients with FTD-*GRN*. The only exceptions to this trend were lower levels of LAMP-2 in patients with Pick’s disease than patients with FTD-*GRN* (Fig. 5b, which still trended higher than controls), and the lack of accumulation of low molecular weight GCase in patients with FTLD-TDP type C.

### Patients with sporadic FTLD-TDP type C or Pick’s disease do not exhibit the increased lysosomal transcript levels observed in patients with FTD-GRN or FTLD-TDP type A

**Fig. 6:**
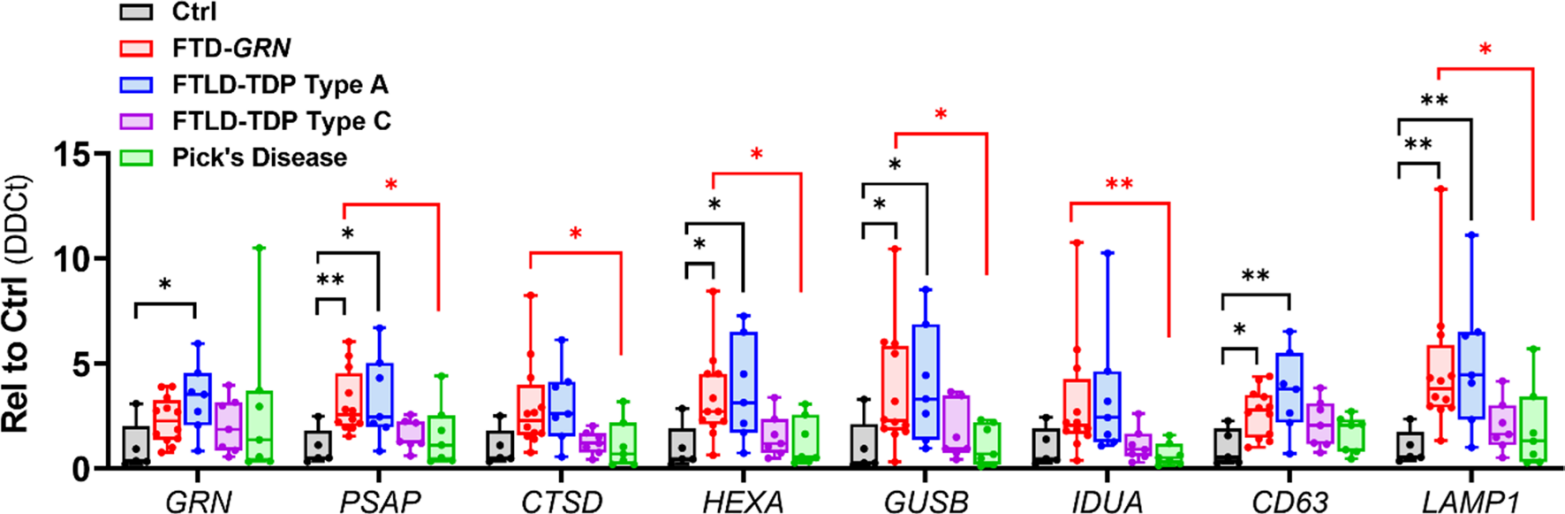
Patients with Sporadic FTLD-TDP Type C or Pick’s Disease Do Not Exhibit the Increased Lysosomal Gene Expression Observed in Patients with FTD-*GRN* or FTLD-TDP Type A. qPCR for a subset of lysosomal transcripts detected by Nanostring analysis (Fig. 2) generally validated their increased expression in frontal cortex of patients with FTD-*GRN* and sporadic FTLD-TDP type A (MANOVA effect of group, *p* = 0.01). However, none of these genes were significantly elevated from control in frontal cortex of patients with FTLD-TDP type C or Pick’s disease. *p < 0.05, **p < 0.01 by Tukey’s post-hoc test. Black lines and symbols indicate difference from control. Red lines and symbols indicate difference from FTD-*GRN*. n = 5 controls, 12 patients with FTD-*GRN*, 7 patients with FTLD-TDP type A, 7 patients with FTLD-TDP type C, and 7 patients with Pick’s disease

In contrast to the generally similar changes in lysosomal proteins, qPCR for a subset of the differentially expressed genes found by Nanostring (Fig. 2) revealed distinct changes in patients with FTD-*GRN* and sporadic FTLD-TDP type A versus the other FTLD subtypes (Fig. 6). qPCR validated the increases in *PSAP*, *HEXA*, *GUSB*, *CD63*, and *LAMP1* for patients with FTD-*GRN* and sporadic FTLD-TDP type A, and in *GRN* for patients with sporadic FTLD-TDP type A (Tukey’s post-hoc test for *GRN* in Ctrl vs. *GRN*, *p* = 0.078). *CTSD* and *IDUA* exhibited statistically non-significant trends for increases versus controls (Tukey’s post-hoc test, *CTSD* Ctrl vs. *GRN*, *p* = 0.056, *CTSD* Ctrl vs FTLD-TDP-A, *p* = 0.089, *IDUA* Ctrl vs. *GRN*, *p* = 0.073, *IDUA* Ctrl vs. FTLD-TDP-A, *p* = 0.065). In contrast, patients with FTLD-TDP type C or Pick’s disease exhibited no significant changes in any of these genes versus controls. Direct comparison between patients with FTD-*GRN* and other FTLD groups revealed that patients with FTD-*GRN* had higher expression of most of these genes than patients with Pick’s disease (Fig. 6, red symbols). Thus, while signs of general lysosomal dysfunction were similar across FTLD subtypes (increases in CatD, LAMP1 and LAMP2, and lysosomal storage material), changes in expression of this subset of lysosomal genes were generally unique to FTLD-TDP type A, regardless of *GRN* status.

## Discussion

In this study, we report that many of the lysosomal abnormalities observed in frontal cortex of patients with FTD-*GRN* [8, 40, 90] are also present in frontal cortex of patients with several types of sporadic FTLD. Patients with sporadic FTLD-TDP types A and C and FTLD-tau (Pick’s disease) exhibited similar increases in CatD activity, LAMP-1 and LAMP-2 levels, and lysosomal storage material as patients with FTD-*GRN*. Though we analyzed a limited number of FTLD subtypes, these data suggest that such lysosomal abnormalities may be a common feature of end-stage FTLD. These lysosomal abnormalities may be associated with multiple aspects of FTLD pathology and neurodegeneration.

The increases in neuronal CatD (Additional file 1: Fig. S1, Fig. 4) and lysosomal storage material (Additional file 1: Fig. S4) are consistent with prior studies of FTD-*GRN* [40, 90] and FTD-*CHMP2B* [28], and may be a sign of neuronal lysosomal dysfunction. Lysosomal dysfunction may be an important contributor to FTLD-TDP and FTLD-tau pathogenesis, as the endolysosomal and autophagy-lysosomal pathways clear TDP-43 and tau, and disruption of these pathways exacerbates both types of pathology [12, 53, 54, 78, 88, 89, 96]. Mutations in several genes involved in autophagy-lysosomal function cause FTLD-TDP [72, 86, 87], implicating lysosomal dysfunction in the initiation of FTLD-TDP. Additionally, both TDP-43 and tau pathology may disrupt lysosomal function, driving further pathology. Loss of nuclear TDP-43 is a key aspect of TDP-43 pathology [4, 65] that may disrupt the autophagy-lysosomal and endolysosomal systems [15, 71, 77, 95]. Pathologic tau can also inhibit autophagy [19, 20], and the FTD-causing R406W tau mutation disrupts lysosomal function in iPSC-derived neurons [60].

While lysosomal dysfunction is implicated in FTLD-TDP and FTLD-tau pathogenesis, the increased CatD and lysosomal storage material in FTLD might also be downstream of neurodegeneration. Similar increases in neuronal cathepsin D and lysosomal storage material have been observed in degenerated regions of brains from patients with Alzheimer’s disease (AD) [21, 22], though these changes are closely associated with AD pathology [23]. Accumulation of lysosomal storage material has also been observed in models of stroke [9], excitotoxicity [48] and traumatic brain injury [45, 47, 70], perhaps indicating a general relationship with neuronal stress or death. In contrast, CatD levels increase in some models of injury and excitotoxicity [11, 13, 16], but decrease in models of hypoxia and stroke [61] and at early time points after traumatic brain injury [74].

Neuroinflammation may also drive some of the lysosomal abnormalities we observed in patients with sporadic FTLD. Degenerated brain regions in FTLD contain many reactive glia [52, 57], which strongly express lysosomal genes [75, 102, 103]. Reactive glia may drive several of the changes we observed, such as increased *GRN* expression in patients with FTD-*GRN* and sporadic FTLD-TDP A (Fig. 2, Additional file 1: S1) [25]. Glial phagocytosis of dead neurons may explain the presence of storage material in glial cells of patients with sporadic FTLD (Additional file 1: Fig. S7), as similar effects have been observed in microglia in rodent models of traumatic brain injury [45, 47, 70].

In summary, the increases in lysosomal proteins and storage material in patients with sporadic FTLD and FTD-*GRN* [8, 40, 90] may be driven by a combination of both dysfunctional neurons and reactive glia. Further investigation will be needed to determine if the increased CatD and storage material in neurons are an indicator of lysosomal dysfunction that may contribute to FTLD pathogenesis or a more non-specific indicator of neuronal distress and death.

This study also raises questions about the relationship between progranulin haploinsufficiency, lysosomal dysfunction, and FTLD-TDP pathology in patients with *GRN* mutations. Comparison of lysosomal measures in frontal and occipital cortices of patients with FTD-*GRN* and sporadic FTLD-TDP type A (Figs. 1, 2, 3, Additional file 1: S1, S2) suggests an association of lysosomal abnormalities with TDP-opathy, neurodegeneration, or inflammation rather than with progranulin haploinsufficiency. Notably, patients with FTD-*GRN* also exhibit lipofuscin accumulation, loss of nuclear TDP-43, and degeneration in the retina [90, 91]. These data may indicate that these lysosomal abnormalities are primarily driven by TDP-opathy, neurodegeneration, or neuroinflammation rather than by progranulin haploinsufficiency. If so, then progranulin haploinsufficiency, unlike complete progranulin deficiency, may produce relatively mild lysosomal dysfunction in the brain. This is also observed in mouse models. Brains from *Grn*^*–/–*^ mice, which model the progranulin deficiency of NCL [2, 44, 82], exhibit robust lysosomal changes and lipofuscinosis [1, 31, 32, 40, 56, 83]. However, brains from *Grn*^+*/–*^ mice, which model the progranulin haploinsufficiency of FTD-*GRN*, exhibit milder changes in lysosomal protein levels [5, 6, 31] and fail to accumulate lipofuscin [1, 32].

Alternatively, the association of lysosomal abnormalities with FTLD-TDP pathology in FTD-*GRN* may indicate regional differences in vulnerability to progranulin haploinsufficiency. Such vulnerability might arise via several mechanisms. In the retina, the daily cycle of photoreceptor degradation places high demand on lysosomes in cells of the retinal pigment epithelium [51], which might make them more vulnerable to disruption by progranulin haploinsufficiency. Within the cortex, regional differences in progranulin cleavage might contribute to selective vulnerability. Greater progranulin cleavage into granulins might contribute to neurodegeneration, as progranulin and granulins have distinct effects on inflammation [108] and lysosomal protease activity [17, 18]. Mouse studies indicate differential cleavage of progranulin between brain regions, with the cortex having a particularly high granulin to progranulin ratio [101]. Patients with FTD-*GRN* exhibit greater cleavage of progranulin to granulins in a degenerated region of frontal cortex than in occipital cortex [63], but this higher cleavage of progranulin may be driven by neurodegeneration or inflammation, as it is also observed in degenerated regions of patients with sporadic FTLD-TDP type A and AD [73].

As in sporadic FTLD, the role of lysosomal dysfunction in FTD-*GRN* pathogenesis will therefore be an important topic for future investigation. The presence of similar lysosomal abnormalities in patients with FTD-*GRN*, patients with sporadic FTLD, and TDP++ mice shows that progranulin haploinsufficiency is not necessary to produce increases in some lysosomal proteins and storage material. However, this does not rule out progranulin haploinsufficiency as an early driver of lysosomal abnormalities in patients with FTD-*GRN*. For example, data from *Grn*^*–/–*^ mice suggest a model in which progranulin deficiency induces lysosomal dysfunction in microglia [39, 56], which then drives neuronal dysfunction and TDP-opathy at later ages [39, 56, 100].

Despite similar signs of lysosomal dysfunction among all FTLD patient groups, patients with FTD-*GRN* and sporadic FTLD-TDP type A exhibited unique changes in expression of lysosomal genes (Figs. 2, 6). Patients with FTLD-TDP type A, regardless of *GRN* genotype, might therefore exhibit unique lysosomal changes compared to other FTLD subtypes. Patients with FTD-*GRN* or sporadic FTLD-TDP type A also exhibit unique transcriptional changes in other pathways, as a recent transcriptomic study showed substantial overlap in differentially-expressed genes among patients with FTD-*GRN* and sporadic FTLD-TDP type A, which diverged from changes in patients with FTLD-TDP type C [68]. Understanding the role of transcriptional dysregulation in FTLD-TDP type A pathogenesis is therefore another important area for future investigation.

## Conclusions

This study shows that patients with several subtypes of sporadic FTLD have similar increases in CatD activity, lysosomal membrane proteins, and lysosomal storage material as patients with FTD-*GRN*. These changes may be driven by lysosomal dysfunction associated with FTLD-TDP or FTLD-tau pathology, or with neurodegeneration and neuroinflammation. In contrast, the unique changes in lysosomal gene expression of patients with FTD-*GRN* and sporadic FTLD-TDP type A indicate lysosomal changes specific to FTLD-TDP type A. These data indicate that lysosomal abnormalities may be a common feature of end-stage FTLD, though they may be driven by distinct mechanisms in different FTLD subtypes.

## Supplementary Information

**Additional file 1:** **Fig. S1.** Quantitation of Low Molecular Weight GCase. **Fig. S2.** Correlation of Triton-insoluble 25 kDa p-TDP-43 with Levels of Lysosomal Proteins. **Fig. S3.** Progranulin, Cathepsin D, and GCase labeling by cell type. **Fig. S4.** Confirmation of TDP-43 aggregation in TDP-43 transgenic mice. **Fig. S5.** Further analysis of storage material in TDP-43 transgenic mice. **Fig. S6.** GCase Levels in Patients with Multiple FTLD Subtypes. **Fig. S7.** Cellular distribution of autofluorescent storage material in frontal cortex.

**Additional file 2:** Tables of patient data.

## Data Availability

All data generated or analyzed during this study are included in this published article and supplemental material.

## Abbreviations

CatD: Cathepsin D
FTD: Frontotemporal dementia
FTD-*GRN*: Frontotemporal dementia due to progranulin mutations
FTLD: Frontotemporal lobar degeneration
GCase: β-Glucocerebrosidase
*GRN*: Progranulin
HexA: β-Hexosaminidase A
NCL: Neuronal ceroid lipofuscinosis
SCMAS: Subunit C of mitochondrial ATP synthase
TDP-43: TAR DNA binding protein of 43 kDa
