# Supplementary material for: Patients with sporadic FTLD exhibit similar increases in lysosomal proteins and storage material as patients with FTD due to GRN mutations
Source: Acta Neuropathol Commun. 2023 Apr 28;11:70. doi: 10.1186/s40478-023-01571-4 (PMC10148425; doi:10.1186/s40478-023-01571-4)
Supplement: Supplementary file 1 — Additional file 1: Fig. S1. Quantitation of Low Molecular Weight GCase. Fig. S2. Correlation of Triton-insoluble 25 kDa p-TDP-43 with Levels of Lysosomal Proteins. Fig. S3. Progranulin, Cathepsin D, and GCase labeling by cell type. Fig. S4. Confirmation of TDP-43 aggregation in TDP-43 transgenic mice. Fig. S5. Further analysis of storage material in TDP-43 transgenic mice. Fig. S6. GCase Levels in Patients with Multiple FTLD Subtypes. Fig. S7. Cellular distribution of autofluorescent storage material in frontal cortex. [file 40478_2023_1571_MOESM1_ESM.pdf]

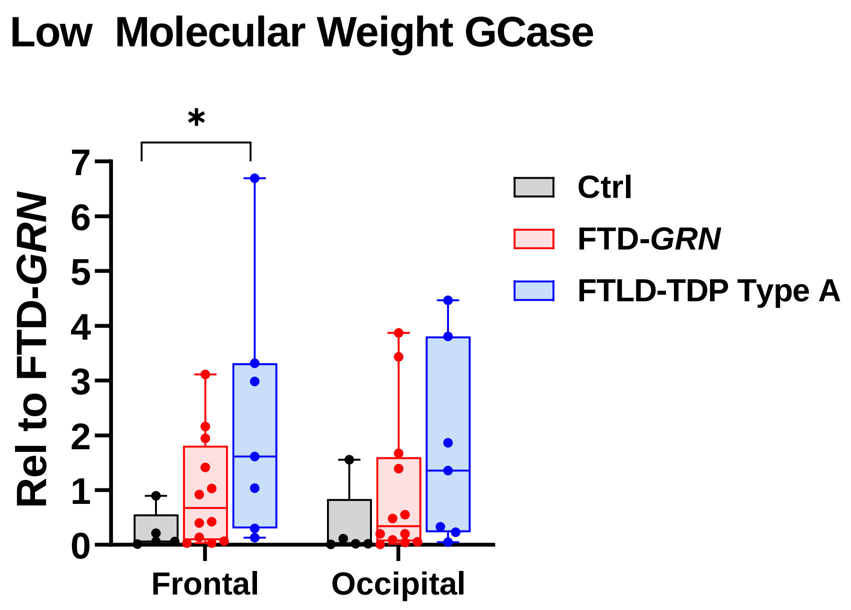

Figure S1 – Quantitation of Low Molecular Weight GCase. Levels of low molecular weight GCase were elevated in frontal cortex of patients with FTLD-TDP type A versus controls (ANOVA, $p = 0.0413$), with a similar non-significant trend in occipital cortex (ANOVA, $p = 0.0755$) cortex. n = 5 controls, 12 patients with FTD-*GRN*, and 7 patients with sporadic FTLD-TDP type A. * = $p < 0.05$ by Fisher's LSD post-hoc test. The low molecular weight GCase band is denoted with a red arrow on the blots in Fig. 1.

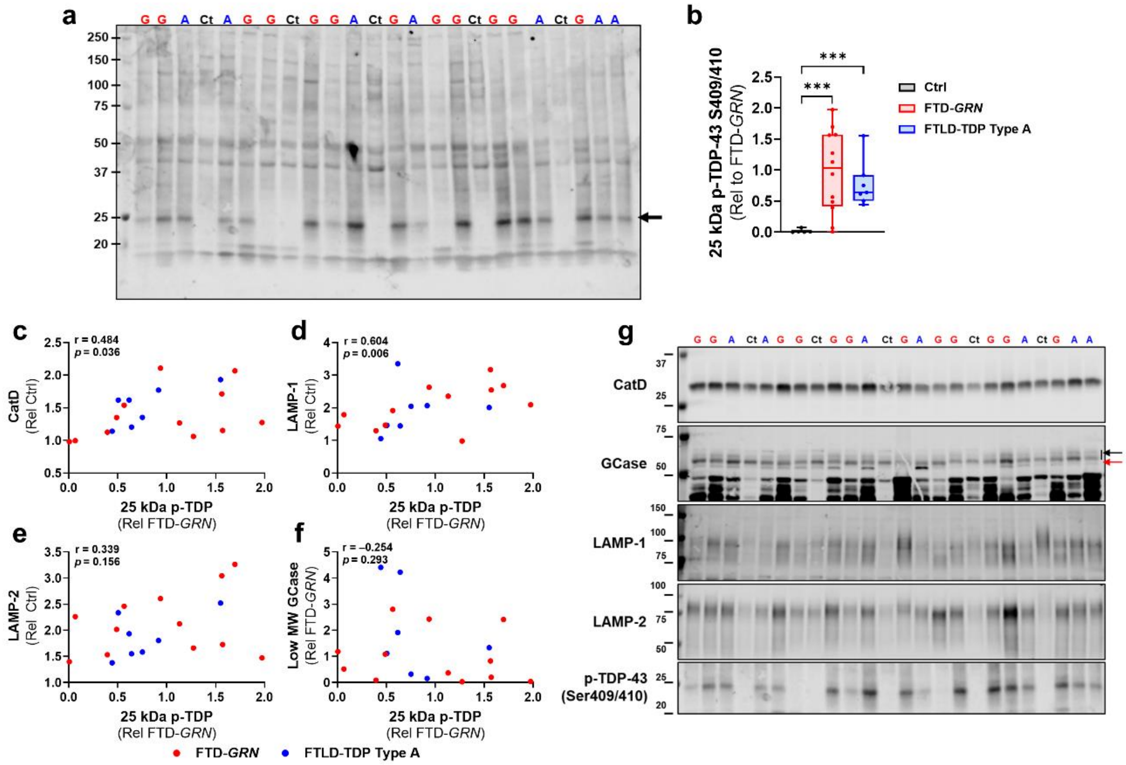

Figure S2 – Correlation of Triton-insoluble 25 kDa p-TDP-43 with Levels of Lysosomal Proteins.

a, Immunoblot of the triton-insoluble pellets of the samples used for assessment of lysosomal enzyme activity and protein levels revealed a roughly 25 kDa p-TDP-43 (Ser409/410) band (shown by arrow). **b**, Levels of 25 kDa p-TDP-43 were significantly higher in patients with FTD-*GRN* or sporadic FTLD-TDP type A than in controls (ANOVA, p = 0.0002), but did not differ between FTLD patient groups. **c–f**, We therefore analyzed the correlation of 25 kDa p-TDP-43 with levels of the lysosomal proteins that were increased in FTLD patient groups. These analyses revealed significant correlation of 25 kDa p-TDP-43 with CatD (**c**) and LAMP-1 (**d**), with a similar trend for LAMP-2 (**e**). Levels of low molecular weight GCase did not exhibit a clear relationship with levels of 25 kDa p-TDP-43 (**f**). In **g**, the 25 kDa p-TDP-43 band is shown aligned with matching CatD, GCase, LAMP-1, and LAMP-2 bands from Fig. 1e. In **a** and **g**, Ct = control, G = FTD-*GRN*, and A = sporadic FTLD-TDP type A. Molecular weight markers are identified by weight in kDa. In **c–f**, Spearman r and p values are shown on each graph. *** = p < 0.001 by Fisher's LSD post-hoc test. n = 5 controls, 12 patients with FTD-*GRN*, and 7 patients with sporadic FTLD-TDP type A.

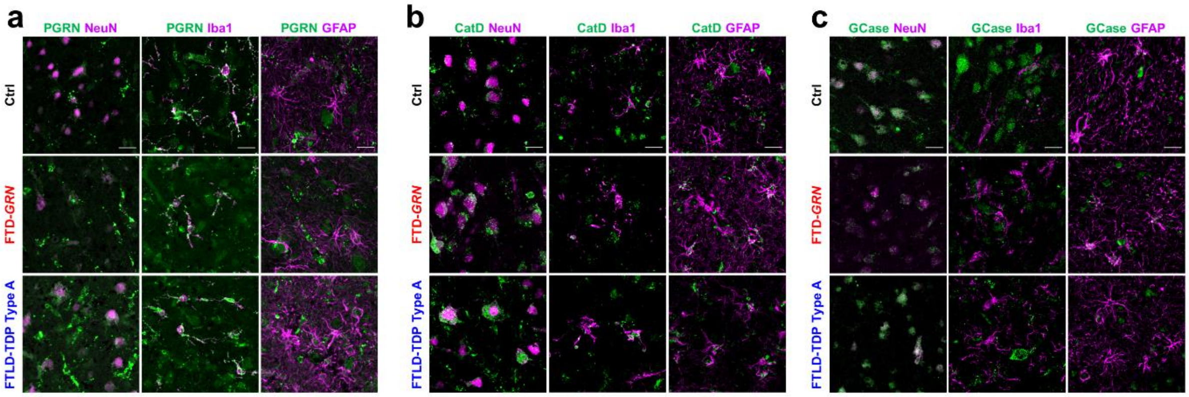

Figure S3 – Progranulin, Cathepsin D, and GCase labeling by cell type. Co-immunostaining for lysosomal proteins (**a**, progranulin (PGRN), **b**, cathepsin D (CatD), and **c** GCase (β-glucocerebrosidase) and markers for neurons (NeuN), microglia (Iba1), and astrocytes (GFAP). All scale bars represent 20 μm.

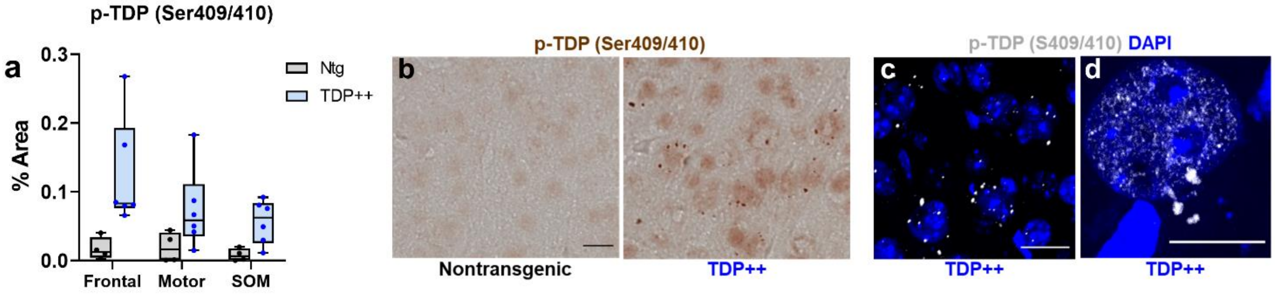

Figure S4 – Confirmation of TDP-43 aggregation in TDP-43 transgenic mice.

a,b, Homozygous TDP-43 transgenic mice (TDP++) exhibited p-TDP-43 (Ser409/410)-immunoreactive aggregates throughout the cortex by 21 days of age (RM ANOVA effect of TDP, *p* = 0.0113, n = 4–6 mice per genotype). **c,d**, Immunofluorescence for p-TDP (Ser409/410) confirmed that these TDP-43 aggregates are cytoplasmic. Scale bars in **b** and **c** repesent 20 μm, and the scale bar in **d** represents 10 μm. SOM = somatosensory cortex.

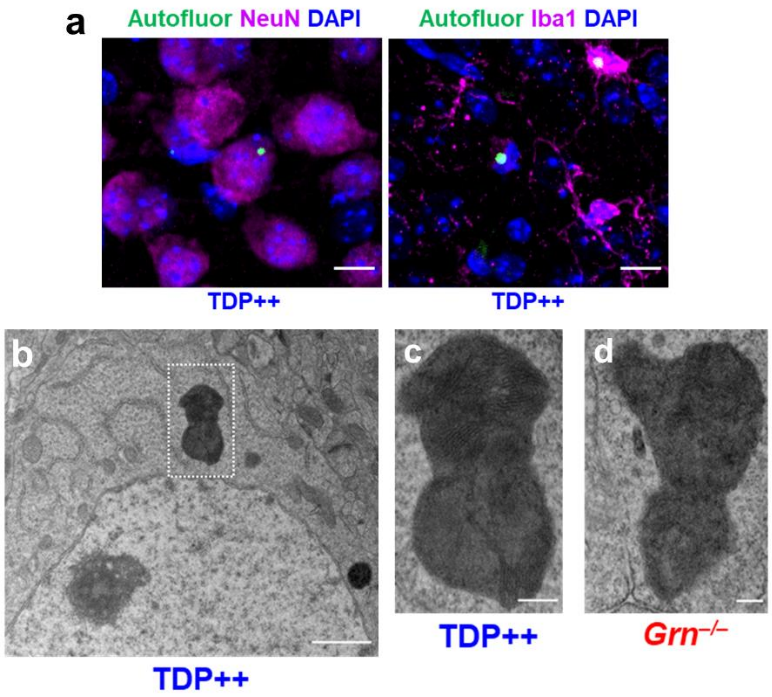

Figure S5 – Further analysis of storage material in TDP-43 transgenic mice.

a, Autofluorescent storage material was present in both neurons and microglia of homozygous TDP-43 transgenic mice (TDP++). **b,c**, Electron microscopy of TDP++ cortex revealed storage deposits with the typical fingerprint profile of lipofuscin [90], **d**, which were similar in appearance to storage material from cortex of *Grn*$^{-/-}$ mice. Scale bars represent 10 µm in **a**, 1 µm in **b**, and 200 nm in **c** and **d**.

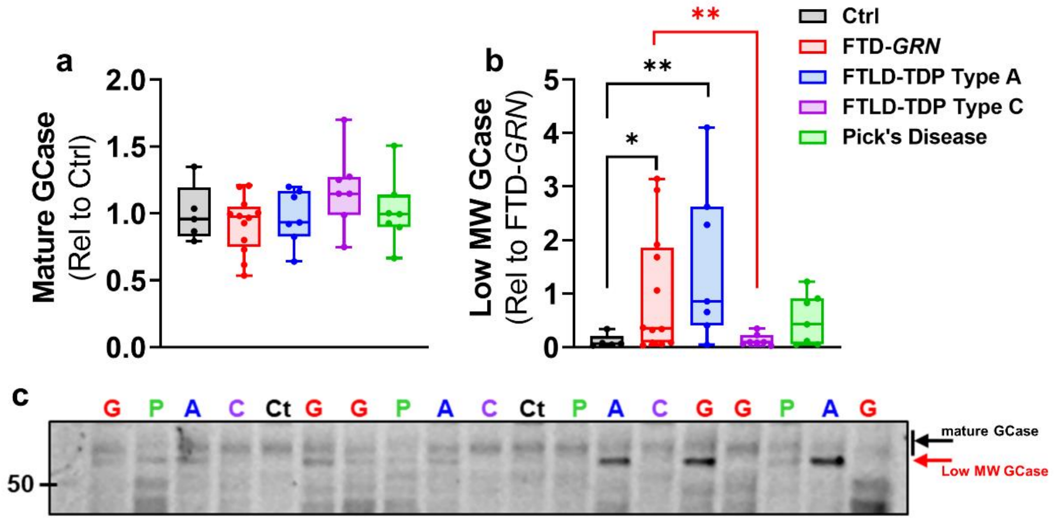

Figure S6 – GCase Levels in Patients with Multiple FTLD Subtypes.

a, GCase immunoblots revealed no significant group differences in mature GCase (ANOVA, p = 0.2567). **b**, Patients with FTD-*GRN* or sporadic FTLD-TDP type A had higher levels of immature, low molecular weight GCase (ANOVA, p = 0.0181). Patients with FTLD-TDP type C did not have higher levels of low molecular weight GCase than controls, and had significantly lower levels of low molecular weight GCase than patients with FTD-*GRN*. Patients with Pick's disease had intermediate levels of low molecular weight GCase, as they did not significantly differ from controls (Fisher's post-hoc test, p = 0.118) but also did not differ from patients with FTD-*GRN*. * = $p < 0.05$, ** = $p < 0.01$ by Fisher's LSD post-hoc test. Black lines and symbols indicate difference from control. Red lines and symbols indicate difference from FTD-*GRN*. Abbreviations for **c** are: Ct = control, G = FTD-*GRN*, A = FTLD-TDP type A, C = FTLD-TDP type C, P = Pick's disease. n = 5 controls, 12 patients with FTD-*GRN*, 7 patients with FTLD-TDP type A, 7 patients with FTLD-TDP type C, and 7 patients with Pick's disease.

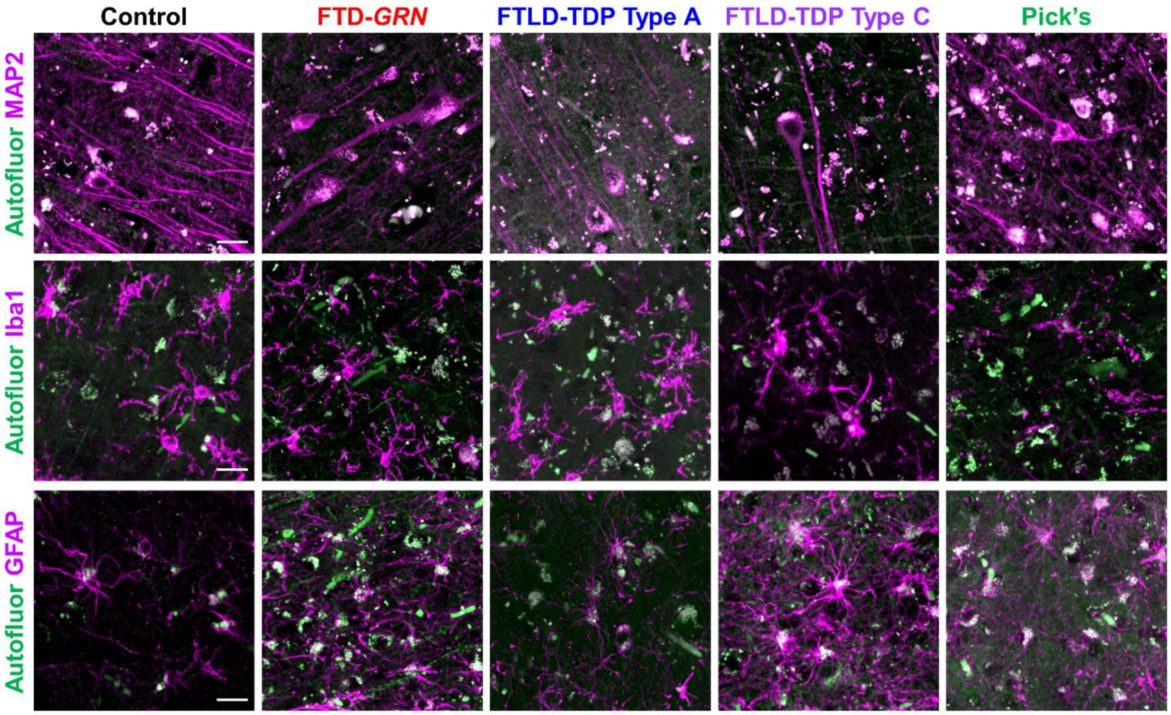

Figure S7 – Cellular distribution of autofluorescent storage material in frontal cortex. Colocalization of autofluorescence and markers of neurons (MAP2), microglia (Iba1), and astrocytes (GFAP) revealed that all cell types carried some autofluorescent storage material. Scale bars represent 20 μm.
